# Mislocalization of Visual Stimuli: Independent Effects of Static and Dynamic Attention

**DOI:** 10.1371/journal.pone.0028371

**Published:** 2011-12-05

**Authors:** Sung-en Chien, Fuminori Ono, Katsumi Watanabe

**Affiliations:** 1 Research Center of Advanced Science and Technology (Cognitive Science), The University of Tokyo, Tokyo, Japan; 2 Japan Science and Technology Agency, Saitama, Japan; Rutgers University, United States of America

## Abstract

Shifts of visual attention cause systematic distortions of the perceived locations of visual objects around the focus of attention. In the attention repulsion effect, the perceived location of a visual target is shifted away from an attention-attracting cue when the cue is presented before the target. Recently it has been found that, if the visual cue is presented after the target, the perceived location of the target shifts toward the location of the following cue. One unanswered question is whether a single mechanism underlies both attentional repulsion and attraction effects. We presented participants with two disks at diagonal locations as visual cues and two vertical lines as targets. Participants were asked to perform a forced-choice task to judge targets' positions. The present study examined whether the magnitude of the repulsion effect and the attraction effect would differ (Experiment 1), whether the two effects would interact (Experiment 2), and whether the location or the dynamic shift of attentional focus would determine the distortions effects (Experiment 3). The results showed that the effect size of the attraction effect was slightly larger than the repulsion effect and the preceding and following cues have independent influences on the perceived positions. The repulsion effect was caused by the location of attnetion and the attraction effect was due to the dynamic shift of attentional focus, suggesting that the underlying mechanisms for the retrospective attraction effect might be different from those for the repulsion effect.

## Introduction

Visual attention can be directed to specific regions other than the fovea. This improves the detection and identification of peripheral visual objects. It also results in better spatial resolution of the attended regions [1]–[3]. Shifts of visual attention to specific locations also cause systematic distortions in perceived locations around the focus of attention, i.e., the attentional repulsion effect [4]. Suzuki and Cavanagh presented two disks as visual cues in diagonally opposite positions (top-left/bottom-right or top-right/bottom-left). Then two vertical lines appeared above and below the center fixation as visual targets. They asked participants to judge horizontal misalignment of two vertical lines. The results showed that the perceived locations of the vertical lines appeared to displace away from the visual cues. That is, if the cues appeared at the top-left/bottom-right position, the participant would be more likely to judge that the top line was on the right side of the bottom line. In the series of Suzuki and Cavanagh's experiments, they also demonstrated that the attentional repulsion effect was not caused by apparent motion or figural aftereffects. They explained this mislocalization as an indication that spatial positions were represented by the overall response patterns of a population of position-coding neural units, such as cells in V1, V2, V3, and V4. The perceived location of the visual stimulus was represented by the centroid of the response distribution of these units. When the attention-attracting cue was presented, attention focused at the cue's location. The target's centroid of response distribution was skewed from the cue, the locus of attention. This could result from surround suppression, recruitment of receptive fields, or shrinkage of receptive fields around the focus of attention. Recent research also showed that the attentional repulsion effect was caused by shifts of attention toward the cued locations [5]–[6]. Another study has indicated that visual attention shifts to the cues' center when the repulsion effect is produced by the onset of the cue, and the magnitude of the repulsion effect depends on the cue-target distance, implying that the effect of attentional shift is not uniform across the visual field [7].

Ono and Watanabe used visual stimuli similar to those used in Suzuki and Cavanagh [4], but the temporal sequence of the stimuli was reversed. In their study, two vertical lines appeared first and were followed by visual cues. They found an attentional attraction effect; if visual cue appeared after the target, the perceived location of the target shifted toward the location of the following cue [8]. Their results showed that attention had a retrospective influence on the spatial perception and the effect was in the direction toward the focus of attention. Different temporal orders of identical visual stimuli can result in distortions of perceived location in opposite directions. They proposed that both repulsion and attraction effect might result from the overshoot of attentional shift, that is, the dynamic attentional shift from the target to the cue shifted beyond cue's actual location. Previous researches of representational momentum and flash-lag tested mislocalization of dynamic targets. They demonstrated that apparent locations were forward displaced due to the overshoot of attention [9]–[10].

One unanswered question is whether a single mechanism underlies both attentional repulsion and attraction effects. In order to investigate this issue, we examined (1) whether the magnitude of the repulsion effect and the attraction effect would differ, (2) whether the two effects would interact, and (3) whether the location or the dynamic shift of attentional focus would determine the distortions effects.

## Experiment 1

Ono and Watanabe examined the attentional attraction effect by using a small number of physical displacements of the target lines [8] and consequently did not report the magnitude of the attraction effect. In Experiment 1, the top line might appear at one of eleven possible positions. We asked participants to judge whether the top line was at the right or left side of the bottom line and estimated the point of subjective equality (PSE), where the proportions of “right response” and “left response” were close to equal.

### Method

#### Ethics Statement

The procedures were approved by the internal review board of Research Center for Advanced Science and Technology, The University of Tokyo, and written informed consent was obtained from all participants prior to the testing.

#### Participants

Fourteen paid volunteers participated. All the participants had normal or corrected-to-normal visual acuity, and were naïve as to the purpose of this study.

#### Stimuli

**Figure 1:**
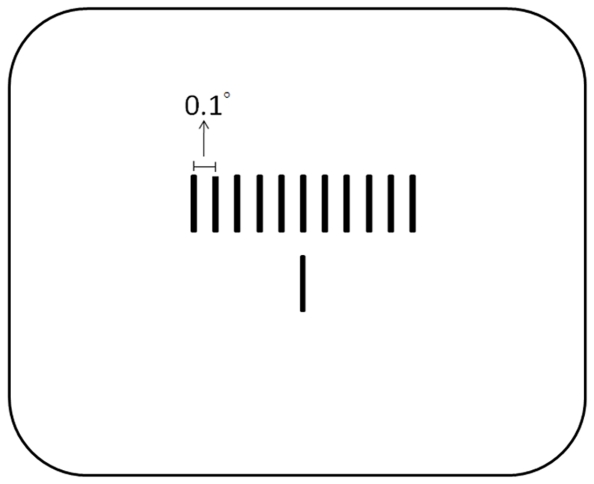
Possible positions for the top line. The top line appeared at one of eleven possible positions. The distance between possible positions was 0.1°. Five were at the left side of the bottom line. The others were at the right side of the bottom line. The leftmost (rightmost) position was 0.5° away from the bottom line in horizontal orientation. The top line was equally likely to appear in one of the eleven positions.

The participants viewed a 17-inch Mitsubushi CRT monitor at a distance of 60 cm. All stimuli appeared in white (69.40 cd/m^2^) against a black (0.01 cd/m^2^) background. The central white fixation point was 0.2° in diameter. The cue stimuli were two disks of 1° in diameter at diagonal locations (i.e., top-left/bottom-right or top-right/bottom-left). The disks were displaced 3.5° in the vertical and horizontal directions from the fixation point. The probabilities of the cues appearing at either diagonal position were the same. The target stimuli were two vertical lines 2.5° above and below the fixation point. Each line was 1.0° long and 0.1° wide. The bottom line appeared just below the location of the fixation point. The top line might appear in one of eleven possible positions (Figure 1). The distance between possible positions was 0.1°. Five were at the left side of the bottom line. The others were at the right side of the bottom line. The leftmost/rightmost position was 0.5° away from the bottom line in horizontal orientation. The top line was equally likely to appear in one of the eleven locations.

#### Procedure

**Figure 2:**
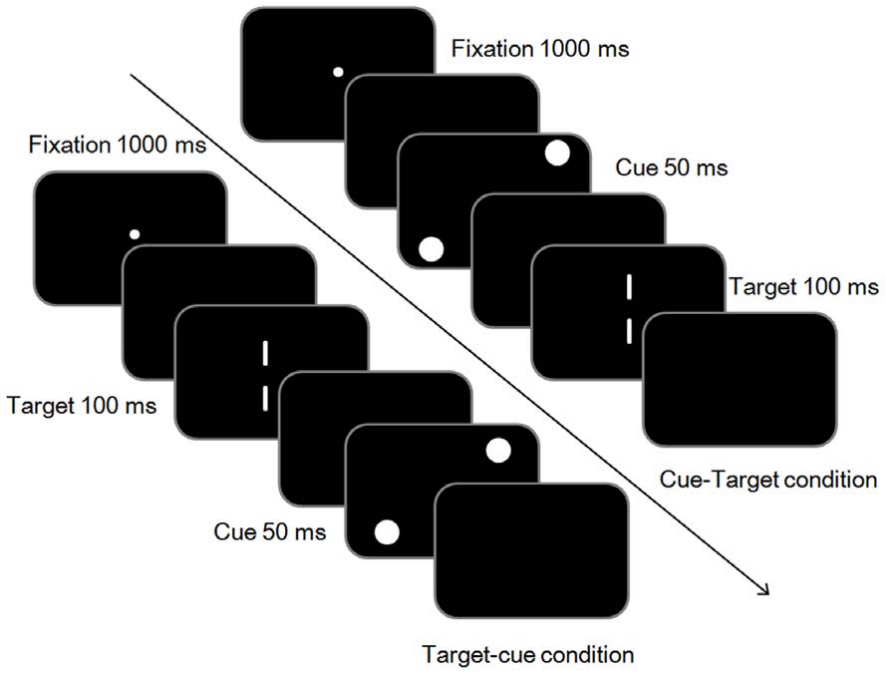
Schematic representation of the trial events in Experiment 1. The fixation point appeared for 1000 ms and participants were instructed to keep their fixation on it. After a 100-ms blank, the cue and target were presented in two different conditions. [1] Under the cue-target condition, the cue was presented for 50 ms. After a 150-ms blank, two vertical target lines were presented for 100 ms. [2] Under the target-cue condition, the target was presented for 100 ms first, followed by a 100-ms blank. Then the cue was presented for 50 ms. The cue-target and target-cue SOAs (stimulus onset asynchrony) were always 200 ms in all conditions.

Participants initiated each trial by pressing the space key. The fixation point appeared for 1000 ms and participants were instructed to keep their fixation on it. After a 100-ms blank, the cue and target were presented in two different conditions. Under the cue-target condition, the cue was presented for 50 ms. After a 150-ms blank, two vertical target lines were presented for 100 ms. Under the target-cue condition, the target was presented for 100 ms first, followed by a 100-ms blank. Then the cue was presented for 50 ms. The cue-target and target-cue SOAs (stimulus onset asynchrony) were always 200 ms in all conditions (Figure 2). This was because previous research showed the attentional repulsion/attraction effect peaked when the cue was presented around 200 ms before/after the target [4], [8]. The two conditions were arranged in a random sequence. Participants were asked to judge whether the top line was located to the left or right of the bottom line by pressing the arrow keys. Even if they perceived that the top and bottom lines were at the same vertical position, they still had to choose one direction (forced-choice task). Each participant practiced 44 times and completed 440 test trials. Although we instructed the participants to keep fixation during entire trial, we did not record their eye movements. However, previous researches indicated that eye movements are not related to both attentional repulsion [5] and attraction effects [9], [11]. Therefore, we thought that the pattern of the results would not change.

### Results and Discussion

**Figure 3:**
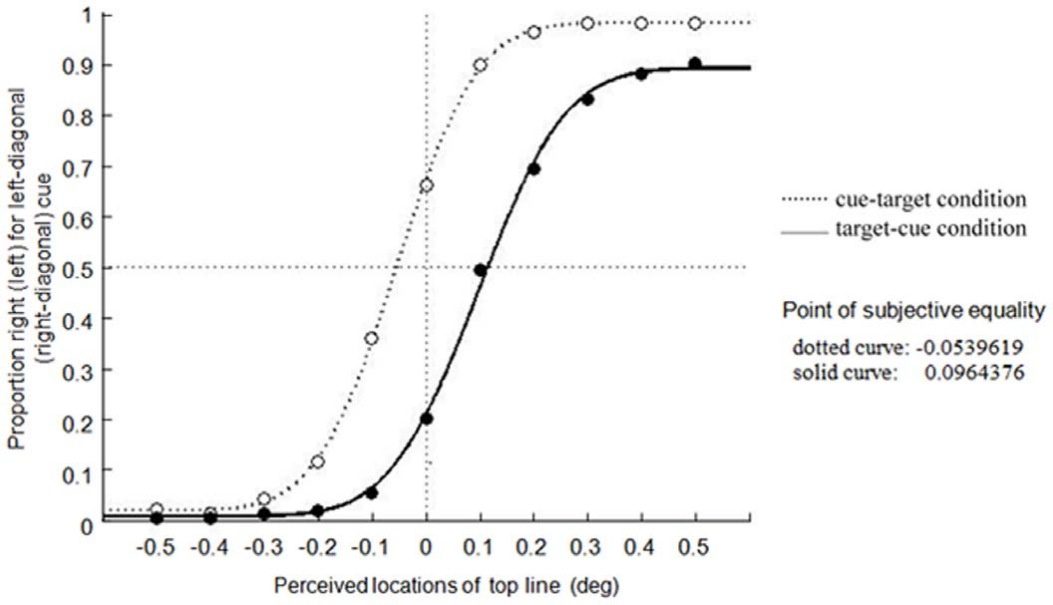
Results of Experiment 1. The vertical axis represents the proportion of “right” response for the left-diagonal cue and “left” response for the right-diagonal cue in each possible target position both under cue-target and target-cue conditions. Positive values on the horizontal axis mean that the top line was at the right of the bottom line for left-diagonal cue and at the left of the bottom line for right-diagonal cue and vice versa. The point of subjective equality (PSE), defined as the intersection of the cumulative Gaussian curves with the line that marked *P* = 0.5, was −0.054° (dotted curve) for the cue-target condition and 0.096° (solid curve) for the target-cue condition.

Figure 3 shows the results of Experiment 1. We calculated the proportion of “right” responses for the left-diagonal cue (top-left/bottom-right) and “left” responses for the right-diagonal (top-right/bottom-left) cue in each possible target position under both cue-target and target-cue conditions. Positive values of the horizontal axis mean that the top line was at the right of the bottom line for the left-diagonal cue and at the left of the bottom line for the right-diagonal cue and vice versa. Note that the proportion of key-press responses in the opposite direction of the diagonal cue was generally lager under the cue-target condition (open circles) than under the target-cue condition (filled circles). The point of subjective equality (PSE), defined as the intersection of the cumulative Gaussian curves with the line that marked *P* = 0.5, was −0.054° (dotted curve) for the cue-target condition and 0.096° (solid curve) for the target-cue condition. The PSEs are the mean of individual PSEs. The coefficient of determination is 0.99 for both conditions in Experiment 1 yielded by the pooled data. The mean of the PSE was significantly smaller in the cue-target condition than the target-cue condition (paired t-test: *t*(13) = 7.36, *p<*.001). The PSEs were significantly different from zero (cue-target condition, *t*(13) = 4.89, *p*<.001; target-cue condition, *t*(13) = 6.18, *p*<.001). In addition, the means of the absolute values of PSEs differed significantly between the cue-target versus target-cue conditions (paired t-test: *t*(13) = 2.25, *p<*.05). These results replicated those of the previous studies [4], [8] and confirmed that the direction of displacement of the target stimuli depended on the timing of the cues. In addition, they showed that the magnitude of spatial distortion was smaller when the cues preceded the target lines than the other way around.

## Experiment 2

The results of Experiment 1 showed that visual cues that followed the target produced spatial distortion with a larger magnitude than preceding cues (although the directions were opposite). In Experiment 2, to examine possible interactions between distortions caused by cues preceding and following the target, we measured spatial distortion when the visual cues were presented both before and after the target. If the preceding and following cues have independent influences on the perceived positions of the target lines, resulting spatial distortion would be a simple sum of repulsion and attraction effects (i.e., the repulsion effect would negate the attraction effect, leaving a smaller attraction effect). On the other hand, it would also be possible that the distortion mechanisms by preceding and following cues are not independent. Then, the spatial distortion would deviate from the simple sum of repulsion and attraction effect.

### Method

#### Participants

Twelve paid volunteers were newly recruited and participated in the experiment. All the participants had normal or corrected-to-normal visual acuity, and were naïve as to the purpose of this study.

#### Stimuli and Procedure

**Figure 4:**
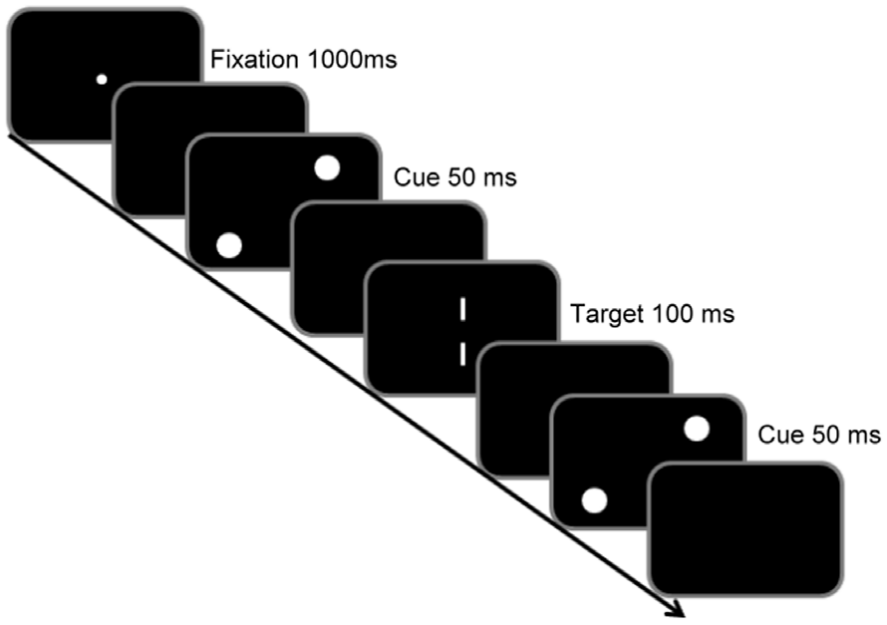
Schematic representation of the trial events in the cue-target-cue condition in Experiment 2. The fixation point appeared for 1000 ms. After a 100-ms blank, the cue and target were presented. The cue was presented for 50 ms. After 150 ms of blank, the target was presented for 100 ms. After another 100 ms of blank, the cue appeared for 50 ms again. The cue-target and target-cue SOA (stimulus onset asynchrony) were always 200 ms.

The apparatus and stimuli were the same as those in Experiment 1. Participants initiated each trial by pressing the space key. The fixation point appeared for 1000 ms and participants were instructed to keep their eyes on it. After a 100-ms blank, the cue and target were presented. The cue appeared two times in each trial and cue was presented for 50 ms. After 150 ms of blank, the target was presented for 100 ms. After another 100 ms of blank, the cue appeared for 50 ms again. The cue-target and target-cue SOA (stimulus onset asynchrony) were always 200 ms (Figure 4). Each participant practiced for 10 times and completed 220 test trials.

### Results and Discussion

**Figure 5:**
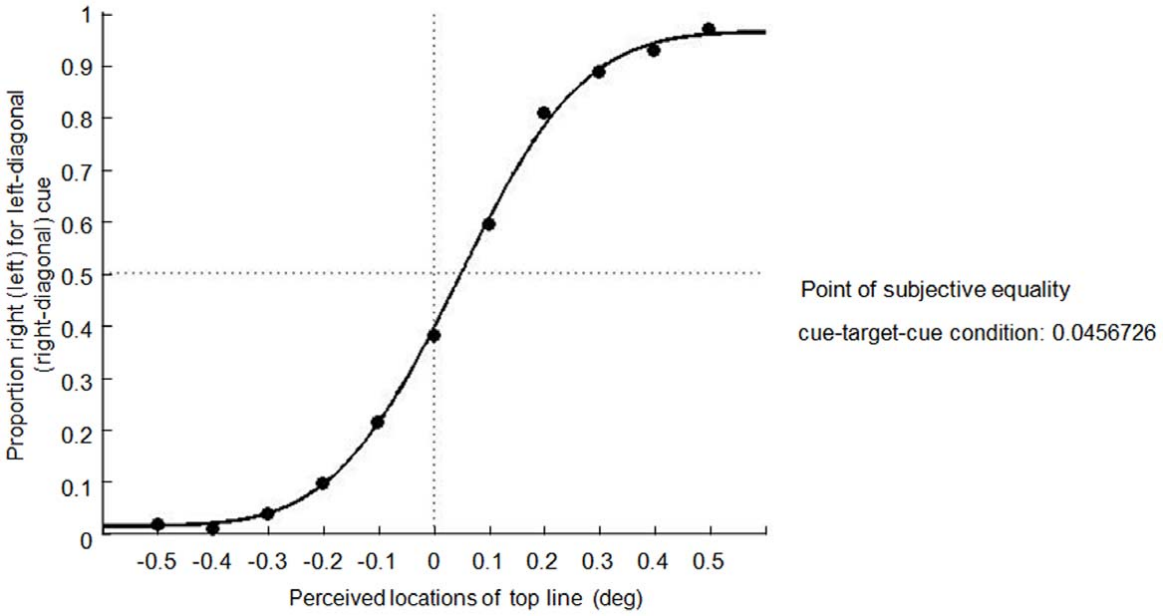
Results of Experiment 2. The mean of PSEs in Experiment 2 was 0.045°. Pooled data provided the data points and fitting curves.

Figure 5 shows the results of Experiment 2. The mean of PSEs, defined as the intersection of the cumulative Gaussian curve with the line that marked *P* = 0.5, was 0.045°. The PSEs are the mean of individual PSEs. The coefficient of determination is 0.99 in Experiment 2 yielded by the pooled data. The spatial distortion observed in Experiment 2 was close to the simple summation of repulsion and attraction effects in Experiment 1 (−0.054°+0.096° = 0.042°). We calculated the sum of repulsion and attraction effects for each participant in Experiment 1 and compared them with those in Experiment 2. There was no statistical difference between them (unpaired t-test: *t*(22) = 0.16, *p* = 0.43). The results thus implied that the perceived location of the target line was influenced independently by both preceding and following cues.

However, the results of Experiment 2 could be explained in other ways. It was also possible that the simple sum was due to each phenomena were processed serially in one single localization mechanism without weighting. Therefore, in Experiment 3, we aimed at further examining whether the repulsion and attraction effect were resulted from different mechanisms.

## Experiment 3

The results of the previous experiments showed that repulsion and attraction effect appeared not to interact but simply to add to each other. Suzuki and Cavanagh considered that repulsion was due to the briefly presented cue attracting visual attention at the beginning of each trial. On the other hand, Ono and Watanabe considered that attraction effect was caused by the overshoot of attentional shift from the target to the cue, which meant that attention shifted beyond cue's actual location. The difference between these two hypotheses was that attraction effect was caused by the dynamic attentional shift but repulsion was caused by attention focused on cues' locations.

**Figure 6:**
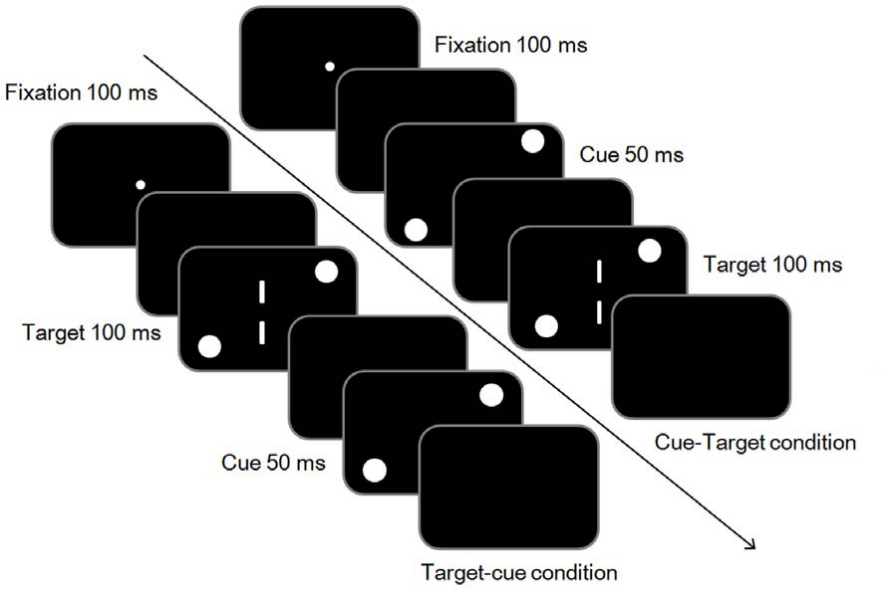
Schematic representation of the trial events of the double-cue condition in Experiment 3. The cues appeared at the same time and duration as the target lines. The cue-target and target-cue SOA were always 200 ms.

In Experiment 3, we presented the cue and target simultaneously in one frame, and the cue was also presented again before or after the target frame (Figure 6). Participants' attention would be attracted to cues' positions at the target frame in either cue-target or target-cue temporal order, at least partially. If the attraction effect was caused by the overshoot of attention from target to cue, the attraction effect under target-cue temporal order would be attenuated in Experiment 3 because not all attention resources focused on the target at the target frame; therefore, the amount of shifted attention resources would be smaller. In contrast, if the repulsion effect under cue-target temporal order was caused by attention focused on the cues' locations, the magnitude would not be affected because attention would be attracted to the cue's location at the cue frame.

### Method

#### Participants

Eleven paid volunteers were newly recruited and participated in the experiment. All the participants had normal or corrected-to-normal visual acuity, and were naïve as to the purpose of this study.

#### Stimuli and Procedure

The top line of targets appeared in one of three locations—directly above (0°rees), to the left of (−0.3°), or to the right of (+0.3°) the bottom line. The cues appeared at the same time and duration as the target lines in half of the trials (double-cue condition). In the other half of trials, the cues appeared only once either before or after targets (single-cue condition). Visual stimuli might be presented in either cue-target or target-cue temporal order. Participants were instructed to perform a forced-choice task to judge whether the top line was located to the left or right of the bottom line. The experiment utilized a 2×2 within-subject design (cue-target versus target-cue×single-cue versus double-cue). The cue-target and target-cue SOA were always 200 ms in all conditions. Participants performed a forced-choice task to judge the perceived location of the top line. Each participant practiced 10 times and completed 240 test trials.

### Results and Discussion

**Figure 7:**
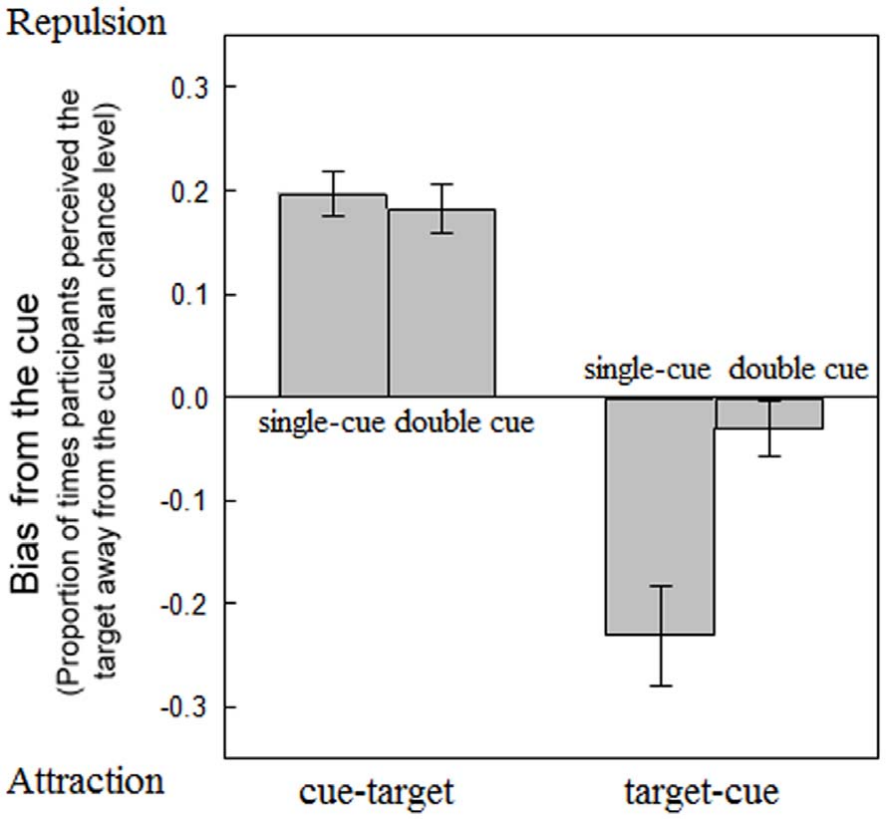
Results of Experiment 3. The positive values on the vertical axis indicated that the perceived location of the target was away from the cue (attentional repulsion effect) and negative value implied that the perceived location of the target was shifted toward the cue (attentional attraction effect). Error bars represented the standard error of mean.

We calculated the averaged “bias away from the cue” to estimate position representation (Figure 7). The bias was computed as the mean of the proportion of “right” response for the left diagonal cue (top-left/bottom-right) and proportion of “left” response for the right diagonal cue (top-right/bottom-left). A positive value indicated that the perceived location of the target was away from the cue (attentional repulsion effect) and a negative value implied that the perceived location of the target was shifted toward the cue (attentional attraction effect).

A two-way ANOVA revealed that main effects of the temporal order (cue-target or target-cue) and the cue manipulation (single or double-cue) were significant [*F*(1, 10) = 84.38, *p*<.001; *F*(1, 10) = 5.78, *p*<.05], and the interaction between the temporal order and the cue manipulation was also significant [*F*(1, 10) = 17.57, *p*<.005]. Post-hoc tests showed that differences in frequency between the single- and double-cue conditions were not significant when the cue preceded the target (*t*(10) = 0.86, *p* = .41, *with Bonferroni correction*). However, when the cue followed the target display, the frequency in the single-cue condition was significantly larger than the double-cue condition (*t*(10) = 3.18, *p*<.05, *with Bonferroni correction*). Furthermore, the frequency of the attraction effect in the double-cue condition was not different from zero (*t*(10) = 1.10, *p* = 0.29). Thus, the simultaneous cue at the moment of the target presentation effectively eliminated the attraction effect, which had virtually no influence on the repulsion effect. Therefore, the results of Experiment 3 supported the possibility of differential processes for the repulsion effect and the attraction effect.

**Figure 8:**
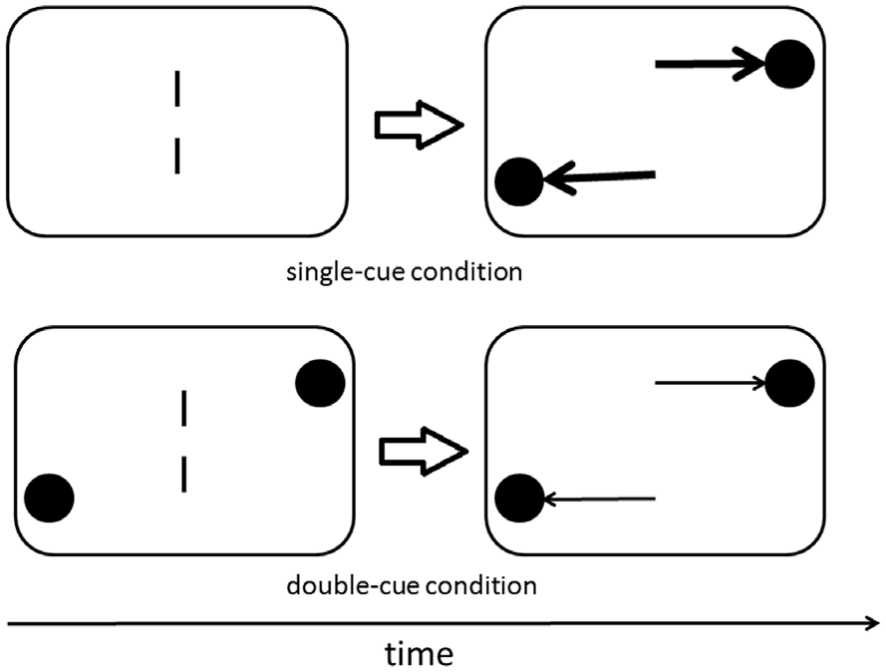
Differential magnitudes of attentional shifts in single-cue and double-cue condition under target-cue temporal order in Experiment 3. Thick and thin arrows represented strong and weak magnitudes of attentional shifts. Shifts of attention were attenuated in double-cue condition due to attention had already been attracted to cues' location at the first presented frame.

In double-cue condition with the target-cue temporal order, visual attention was attracted to both cues' and targets' locations at the beginning of each trial. Thereby, in the next frame, participants did not have to shift visual attention to cues' locations. However, in the single-cue condition with the same temporal order, the participants would shift attention to cues' locations in the second frame because there were no cues in the first frame; hence the attraction effect would occur by the overshoot of attentional shift from the target to the cue [8]. So we did not observe attraction effect in the single-cue condition. On the other hand, we observed the repulsion effect in the double-cue condition with the cue-target temporal order (Figure 8). This can be explained in that the brief cue attracted visual attention at the beginning of each trial [4]. The magnitude of attentional shift from the cue to the target was attenuated, but it had no influence on repulsion effect.

## Discussion

In order to investigate the extent to which the repulsion effect by preceding cues and the attraction effect by following cues share underlying mechanisms, the present study tested whether the magnitude of the repulsion effect and the attraction effect would differ (Experiment 1), whether the two effects would interact or simply add up when the cue was presented both before and after the target (Experiment 2), and whether the cue that is simultaneously presented with the target would similarly influence the repulsion effect and the attraction effect (Experiment 3). The magnitudes of the repulsion and attraction effect were −0.054° and 0.096°, respectively, in Experiment 1. In Experiment 2, we found that the effect size of the attraction effect was close to the simple summation of repulsion and attraction effects in Experiment 1 when the cue was presented both before and after the target. Experiment 3 indicated that the simultaneous cue at the timing of the target diminished the attraction effect but had no influence on the repulsion effect. Overall, the present results suggest that the underlying mechanisms for the retrospective attraction effect might be partially different from those for the repulsion effect. We conjecture that static attention induces the repulsion effect and dynamic attention induces the attraction effect. Static and dynamic attention both had influences on localization mechanism. But both of them might not be necessary to affect the localization mechanism simultaneously. Suzuki and Cavanagh [4] posited the hypothesis that the repulsion effect is one of the costs of a general mechanism that operated to enhance perception at an attended location. According to their position-coding hypothesis, the perceived location is represented by the centroid of the distribution of position-coding units. Attention is directed to the location where peripheral cues are presented. This attentional shift would cause the centroid of distribution shift to the opposite direction toward the direction of attentional shift. They proposed that this mislocalization might be due to surround suppression, receptive field recruitment, or receptive field shrinkage. Receptive field shrinkage predicts that the perceived location of a visual target is always repelled from the cue. However, the hypothesis of surround suppression with receptive field recruitment also predicted attraction effect even when the cue-target distance was closer. But, repulsion did turn to attraction effect when the cue-target distance was less than 20–30 min (visual angle) in the previous study [4]. Recent research has indicated that visual attention shifts to the center of visual cues in the repulsion effect [7], supporting the position-coding hypothesis. However, this hypothesis is not consistent with the dependency of spatial distortion on whether the cue was presented before or after the target.

Other research has indicated that visual receptive fields in the primate middle temporal area dynamically shift in the direction of attentional shift, increasing selectivity of visual representations within and across the visual area [12]. This might explain the attraction effect, that is, the overshoot of attentional shift from the target to the cue displaced the perceived location of the target toward the location of the cue [8].

The results of Experiments 3 point to differential mechanisms for the repulsion and attraction effects. The simultaneous cue at the moment of target affected only the attraction effect. Under the double-cue condition with the target-cue temporal order, both cue and target were presented simultaneously at the beginning of each trial. Both of them should attract visual attention. When cues were presented alone, attention resources that were distributed to the target shifted to the cues' locations. Comparing the double-cue to the single-cue condition, the magnitude of attentional shift would be larger in the single-cue condition because only the target attracted attention at the moment of target presentation. The attenuated shift of attention under the double-cue condition might diminish the attraction effect, supporting the idea that attraction effect was caused by the overshoot of attentional shift from the cue to the target. However, the attenuated shift of attention from the cue to the target did not diminish the repulsion effect. Therefore, it might be speculated that the repulsion effect is due to the shift of the centroid of the distribution of position-coding units, whereas the attraction effect involves dynamic shift of visual attention. Future study further investigations are warranted for examining these possibilities.

What might be the possible mechanism for the attraction effect? Compression of visual space toward the saccade target had been observed in experiments using briefly flashed stimuli [13]. Recent modeling studies indicated that this compression resulted from a spatially selective feedback signal encoding saccade signal. It was used to boost visual performance around the saccade target transiently by increasing the gain of cells with receptive fields around the target. Compression is the cost of this improvement. Covert shifts of attention could be taken as motor plans to move the eyes. This might be sufficient to cause compression of visual space [14]–[15]. Compression of visual space could be the cause of attraction effect resulting from dynamic shifts of attention. When participants shift attention from targets to cues, visual space is compressed toward the cues. This effect also might retrospectively influence location representations of previously presented visual objects; thus, their perceived locations might be shifted toward the cues. But compression due to dynamic shifts of attention cannot explain the repulsion effect. If visual space is compressed when participants shift attention from the cues to the targets, visual space should be compressed toward target locations; participants would perceive targets' apparent locations toward their physical locations, not repelling from cues' locations.

Suzuki and Cavanagh's experiments had already shown that the repulsion effect could not be attributed to apparent motion [4]. They demonstrated that repulsion occured even when apparent motion went in the opposite direction. However, apparent motion could be an alternative explanation for the attraction effect. Sequential presentation of static objects in different positions could induce apparent motion, so there was a possibility that our static visual stimuli presented at different locations induced apparent motion between cue and target. In experiments containing following cues, the direction of motion signals was toward the peripheral cues. According to the motion-biasing model, perceived locations would be biased in the direction of motion because the visual system accounts for neural processing delays by pushing an object close to its physical location retrospectively [16] and the perceived target locations are shifted toward cues. In Experiment 3, the diminished attraction effect in the target-cue temporal order could be explained as evidence that the quality of apparent motion was impaired by the simultaneous cue. Therefore, it is possible that the different mechanisms inducing repulsion and attraction effects in this study are static attention focusing at cue's location and apparent motion, respectively. However, Ono and Watanabe's studies indicated that if the left and right diagonal cues were presented simultaneously, the attraction effect occurred only when participants paid attention to specified cues. This implied that even tough the attraction effect might be induced by apparent motion, attention was still required to select the direction of apparent motion.

Influences of visual landmarks have been also used to explain perceptual mislocalizations of visual objects. Apparent locations of visual stimuli are shifted toward the landmark in the visual field [17]–[18]. The effect of a landmark seems to result from the bias of short-term memory trace, which could also be explained by attention [11], [16], [19]. That is, cue stimuli attract attention that modulates the averaging of location information between objects in short-term memory. It might help to explain the attraction effect. With the target-cue temporal order in the present study, the cue was taken as a landmark because participants' task was localization of the target. However, the influence of landmarks on visual localization might not fit well with the repulsion effect. If the mislocalization was caused by participants taking the cues as landmarks, we should also have observed the attraction effect when the preceding cue was presented.

In conclusion, we proposed two partially different mechanisms accounting for the repulsion effect and the attraction effect. Static attention focusing at a cue's location induces repulsion because the centroid of the distribution of position-coding units shifts to the opposite direction toward the attention-attracting cue. Dynamic attentional shift from target to cue causes attraction effect. This might be resulted from either the cost of incremental gain of cells with receptive fields around the following cue or direction of apparent motion selected by visual attention. However, further investigations are warranted for examining these possibilities.
